# Analyzing the complex pathways of cultural capital influencing health from configurational perspective: evidence from China

**DOI:** 10.3389/fpubh.2025.1673171

**Published:** 2025-11-12

**Authors:** Wei Lu, Chongqi Hao, Yan Xuan, Xiaojun Huang, Li Jing

**Affiliations:** 1 School of Public Health, Hainan Medical University, Haikou, China; 2 Hainan Women and Children’s Medical Center, Haikou, China; 3 School of Public Health, Shanxi Medical University, Taiyuan, Shanxi, China; 4 Hainan Open University, Haikou, China

**Keywords:** cultural capital, health, qualitative comparative analysis, social determinants of health, China

## Abstract

**Background:**

Research on the health effects of cultural capital has emerged as a prominent topic in public health. Traditional regression modeling makes it difficult to deal with the complex and diverse relationships between cultural capital and health. In this study, we innovatively introduce the QCA method to reveal the nonlinear mechanisms and synergistic pathways through which individual cultural capital impacts health.

**Methods:**

Using data from Chinese General Social Survey, fsQCA was conducted to identify necessary and sufficient conditions for health outcomes. Variables included institutionalized cultural capital (education), embodied cultural capital (cultural activities), income, exercise frequency, age, gender, marital.

**Results:**

None of the seven variables were necessary to produce a healthy outcome (consistency < 0.9). Nine configurations emerged as sufficient conditions (solution consistency = 0.882 > 0.8). Configurations were categorized into three types: coexistence (configuration 1), single existence (configuration 2–7), non-existence (configuration 8, 9). In coexistence type, high levels of institutionalized cultural capital and embodied cultural capital need to combine with low levels of age and marriage, and high levels of exercise to generate healthy outcome. In Single existence type embodied cultural capital assumes a critical role. In non-existence type, both cultural capital variables become irrelevant, and even the institutionalized cultural capital appears at low level.

**Conclusion:**

Cultural capital influences health through multiple, equifinal pathways rather than in isolation. Embodied cultural capital is a more potent and consistent driver of good health than institutionalized cultural capital in many pathways. Socioeconomic and demographic factors interact with cultural capital, and can sometimes compensate for its lack.

## Background

Global health inequalities continue to intensify. As a core dimension of social stratification, research on the health effects of cultural capital has emerged as a prominent topic in public health. Characterized by multidimensionality, cumulative nature, and intergenerational transmission, cultural capital exerts unique and complex influences on health (1). Numerous studies have demonstrated that cultural capital exerts positive effects on individual health by promoting health-related behaviors and facilitating resource acquisition. However, some investigations suggest that the health impact of cultural capital may be attenuated due to population heterogeneity (2). Furthermore, some investigations reveal that cultural capital might negatively impact health through psychological stress pathways (3). This complex diversity stems from the necessity of synergistic combinations between cultural capital and multiple covariates to generate health outcomes. These interrelated variables (including cultural capital) exhibit interdependent relationships where their varying states (presence/absence) may differentially influence health outcomes (4). Most empirical studies use regression modeling (5), a method that assumes independence among variables and symmetry of results in advance, making it difficult to deal with the complex and diverse relationships between cultural capital and health. Qualitative comparative analysis (QCA), a methodology specifically designed for complex system analysis, demonstrates exceptional capability in examining multiple conjunctural causation and conditional configuration pathways, having gained substantial traction across diverse research domains (6).

In this study, we focus on the relationship between individual-level cultural capital and health, and introduce the QCA method to address two key questions: (1) Is the effect of cultural capital on health positive or negative? (2) Through what pathways does cultural capital influence health outcomes?

### Cultural capital

Cultural capital, as conceptualized by Bourdieu, refers to the cultural resources possessed by individuals that can be transformed into social advantages, encompassing knowledge, skills, cultural competencies, and institutional credentials (7, 8). To further investigate cultural capital’s role in social structures, Bourdieu categorized it into three types: institutionalized cultural capital, embodied cultural capital, and objectified cultural capital.

Institutionalized cultural capital refers to educational attainments (9), such as academic credentials and professional certifications, which symbolize socially validated markers of an individual’s knowledge and competencies. These institutionalized “cultural credentials” codify personal cultural acquisitions, with advanced education and multiple certifications indicating higher levels of institutional cultural capital. Embodied cultural capital manifests as internalized cultural attributes (10), including values, preferences, behavioral norms, and operational skills. Such traits are gradually accumulated through cultural attitudes and social practices, such as attending concerts, visiting museums, watching films, or participating in artistic performances. Objectified cultural capital comprises materialized cultural products (11), such as privately owned artworks and book collections. Possession of greater quantities of these cultural artifacts signifies superior conditions for cultural enrichment and knowledge accumulation, reflecting higher levels of objectified cultural capital. Based on this classification, researchers may quantify cultural capital through survey instruments and explore its associations with health outcomes.

### Cultural capital and health

This study conceptualizes health not merely as the absence of disease, but as a holistic state of complete physical, mental, and social well-being. This conception aligns with the biopsychosocial model, and further indicates that social factors exert a significant impact on health (12). Cultural capital may be associated with socioeconomic and demographic factors, yet it represents unique cultural resources and is capable of exerting an impact on health through specific mechanisms. In this study, we focus on the influence pathways of individual cultural capital on health.

In the area of individual cultural capital, most research confirms the positive health effects of cultural capital. Pinxten and Lievens (13) confirmed the positive impact of cultural capital on physical health. By comparing cultural activities with other leisure pursuits, they demonstrated that it is the cultural elements embedded in cultural engagement—not the physical act of participation—that influence health outcomes. Christensen and Carpiano (14) identified a significant negative correlation between cultural capital and body mass index among women, indicating reduced obesity risks with higher cultural capital accumulation. Kivimäki’s (15) research revealed that educational capital enhances health literacy while reducing high-risk behaviors like smoking and excessive drinking. Li′s (16) study suggested cultural participation alleviates anxiety symptoms through strengthened social connections. However, some studies indicate this promotive effect may weaken across different populations. For instance, although educational expansion increased higher education accessibility, persistent inequalities in cultural capital accumulation have hindered health improvement among disadvantaged groups, potentially exacerbating health disparities (17, 18). Notably, certain research identifies negative health impacts of cultural capital: excessive health standard pursuit among high-cultural-capital groups may induce anxiety (19), while proliferation of vulgar cultural products indirectly harms health through promoting unhealthy lifestyles (20).

Previous studies have revealed that the health effects of cultural capital are characterized by complexity and diversity, which traditional regression analysis methods are ill-equipped to adequately capture. For social capital—another construct similarly marked by these features—Carlota Quintal first applied QCA to explore its complex relationship with health outcomes in European populations (21). Building on this foundation, Hao et al. (22) conducted a QCA-based analysis of Chinese samples. Collectively, these studies have demonstrated the feasibility of QCA for analyzing the social determinants of health.

Building upon prior research, this study adopts a configurational perspective to innovatively introduce the QCA method into the investigation of individual cultural capital, thereby revealing the nonlinear mechanisms and synergistic pathways through which cultural capital impacts health outcomes. The research not only enriches methodological approaches in cultural capital studies but also provides valuable references for the design of differentiated health policies.

## Materials and methods

### Data sources

The data utilized in this study were obtained from the latest release of the Chinese General Social Survey (CGSS) 2021. As China’s earliest national, comprehensive, and continuous academic survey project, CGSS is administered by the National Survey Research Center at Renmin University of China, with financial support from the Scientific Research Fund of Renmin University of China. This cross-sectional survey systematically collects multilevel data encompassing social, community, household, and individual dimensions, aiming to establish a dynamic monitoring framework for social transformation and advance empirical research on critical societal issues (23).

The most recently published data (2021 survey) became publicly accessible in 2023, representing the 14th annual iteration of CGSS. Employing multistage stratified probability-proportional-to-size (PPS) sampling methodology, the 2021 wave successfully collected 8,148 valid samples nationwide (24). Extensively applied across sociological, economic, demographic, and political science research domains, CGSS has facilitated over 1,000 peer-reviewed publications, demonstrating high representativeness and scientific rigor in studies involving Chinese population (25). The survey had received ethical approval from the Biomedical Ethics Committee of Renmin University of China. All participants completed informed consent procedures through oral or written documentation, with subsequent anonymization of questionnaire data.

### Outcome variable

We choose “self-rated health” as an indicator of individual health. As a comprehensive indicator, self-rated health effectively reflects individuals’ holistic perception of health and demonstrates significant correlations with objective health metrics such as morbidity and mortality (26, 27). In studies examining the impact of social determinants on health, self-rated health has been adopted by the majority of researchers as the primary outcome measure (28).

This variable is coded as A15 in the database, corresponding to the survey question: “How would you describe your current physical health status?” Response options were scaled as: 1 = Very unhealthy, 2 = Relatively unhealthy, 3 = Average, 4 = Relatively healthy, 5 = Very healthy.

### Core conditional variables

Given the absence of objective cultural capital measures in the CGSS (29), institutionalized cultural capital and embodied cultural capital were adopted as proxy indicators. Educational attainment (coded A7a) was selected to represent institutionalized cultural capital, corresponding to the question: “What is your highest level of education completed?” Response options are coded as follows: 1 = No formal education, 2 = Private tutoring/literacy classes, 3 = Primary school, 4 = Junior high school, 5 = Vocational senior high school, 6 = General senior high school, 7 = Technical secondary school, 8 = Vocational training school, 9 = Junior college (Adult higher education), 10 = Junior college (Regular higher education), 11 = Bachelor’s degree (Adult higher education), 12 = Bachelor’s degree (Regular higher education), 13 = Postgraduate and above.

Embodied cultural capital was operationalized through eight cultural participation items (A30_1, A30_2, A30_4, A30_5, A30_8, A30_10, A30_11, A30_12). These items shared the common stem: “How frequently did you engage in the following leisure activities during the past year?” covering: (1) Television/DVD viewing; (2) Cinema attendance; (3) Reading (books/newspapers/magazines); (4) Cultural event participation (concerts, exhibitions); (5) Home music listening; (6) Live sports spectatorship; (7) Handicrafts engagement; (8) Internet use. All items employed a 5-point frequency scale: 1 = Daily, 2 = Weekly, 3 = Monthly, 4 = Yearly or less, 5 = Never.

### Additional conditional variables

Consistent with prior research indicating the mediating roles of economic factors and lifestyle in the cultural capital-health relationship, two mediators were included. Personal annual income (A8a) measured economic status through the question: “What was your total personal income in 2020?” Health-related lifestyle was represented by physical exercise frequency (A30_9): “How often did you engage in physical exercise during leisure time last year?” using the same 5-point scale as cultural activities. In addition, we incorporated control variables commonly used in previous studies as conditioning variables. They were age (continuous, A3_1), gender (dichotomous: 1 = Male, 2 = Female; A2), and marital status (A69) with seven categories: 1 = Never married, 2 = Cohabiting, 3 = First marriage with spouse, 4 = Remarried with spouse, 5 = Separated, 6 = Divorced, 7 = Widowed.

### Data reorganization

This study employed STATA 17 for data processing. First, variables were selected based on the aforementioned coding scheme. Subsequently, samples containing responses such as “do not know,” “refuse to answer,” “not applicable,” or “missing” in any variable were excluded, resulting in a final sample size of 5,769 cases. Finally, the variables needed to be reassigned. The assignment results of each variable are shown in Table 1. The assignment process is described in detail below: (1) The self-rated health variable retained its original values without modification. (2) To address the redundant educational classifications in the CGSS dataset, educational attainment was consolidated: original options 1–3 were categorized as Primary school or below, options 5–6 as Senior high school, options 7–8 as Secondary specialized school/Vocational school, options 9–10 as Junior college, and options 11–12 as Bachelor’s degree. (3) Regarding the fact that Embodied cultural capital consisted of 8 survey questions, Bygren et al. (30) transformed each question option into a binary classification (0 and 1) and then summed them up to obtain the total score. Considering that this classification method would result in a significant loss of information, we took the following steps: First, each option was assigned a score. Based on the logic that higher frequency corresponded to a higher score, the 5 options were assigned scores of 4, 3, 2, 1, and 0 in sequence. Then, the scores of the 8 questions were summed up, and the total score was used as the value of Embodied cultural capital (ranging from 0 to 32). (4) Annual total personal income was an absolute number with a large difference. To avoid the adverse impact of extreme values on the model results, we took the logarithm of the income (ln[value+1]). (5) Physical exercise frequency was reverse-coded, assigning original options 1–5 scores 5–1 to align higher scores with greater activity frequency. (6) Among other conditional variables, marriage needs to be explained. We divided marriage into two categories: “Married” included “cohabiting,” “first marriage with spouse,” and “remarried with spouse”; “Unmarried” included “never married,” “separated but not divorced,” “divorced,” and “widowed.”

**Table 1:** Variable assignment and calibration points.

Variable names		Assignment	Calibration		
			Fully in	Crossover point	Fully out
Outcome Variable	Self-rated health	1 = Very unhealthy; 2 = Relatively unhealthy; 3 = Average; 4 = Relatively healthy; 5 = Very healthy	5	3	1
Conditional variables	Age	Value of age at the time of the survey (2021 minus birth year)	74 (90%)	54 (50%)	29 (10%)
	Gender	0 = Female; 1 = Male	1		0
	Marriage	0 = Unmarried (never married, separated but not divorced, divorced, widowed); 1 = Married (cohabiting, first marriage with spouse, remarried with spouse)	1		0
	Income	Logarithm of original income value	11 (90%)	10 (50%)	8 (10%)
	Exercise	1 = Never; 2 = Several times a year or less; 3 = Several times a month; 4 = Several times a week; 5 = Daily	5	3	1
	Institutionalized cultural capital	1 = Primary school or below; 2 = Junior high school; 3 = Senior high school; 4 = Secondary specialized school/Vocational school; 5 = Junior college; 6 = Bachelor’s degree; 7 = Master’s degree or higher	6	4	2
	Embodied cultural capital	Calculated score (0–32)	16 (90%)	10 (50%)	4 (10%)

## Method

QCA operates under the guidance of holistic philosophy, utilizing set theory and Boolean algebra operations to reveal complex causal relationships between multiple condition combinations and outcomes (31). Depending on variable value types, QCA primarily consists of crisp-set (csQCA), multi-value (mvQCA), and fuzzy-set (fsQCA) variants. The fuzzy-set method significantly enhances analytical capability for complex social phenomena through 0–1 continuous membership score calibration.

QCA relaxes the assumptions underlying traditional regression analysis and proposes three key assumptions suitable for complex causal analysis: conjunctural causation, equifinality, and asymmetry. (1) QCA no longer assumes that causal conditions are independent. Thus, it abandons the univariate “net effect” analysis which relies on the assumptions of “additivity” and variable separability. Consequently, QCA is capable of analyzing the interdependencies among causal conditions and the multiple conjunctural causation formed by different combinations. (2) Equifinality refers to the phenomenon that multiple paths can lead to the same outcome. This implies that there are various possible paths to achieve a desired outcome or undesired outcome, and there is no single optimal and balanced path solution as posited in traditional analytical methods. This equifinality can be categorized into two types of configurations: one type consists of multiple configurations with different core conditions; the other type includes multiple configurations sharing the same core conditions but exhibiting equivalent neutral permutations due to differences in peripheral conditions (32). (3) The asymmetric assumptions of QCA can be divided into two types: causal asymmetry and asymmetry in conditional effects (33). Causal asymmetry means that the causes for the occurrence of a desired outcome (i.e., health in this study) are different from those for its non-occurrence (i.e., poor health in this study). Taking this study as an example, if high-level cultural capital is identified as a cause of health, it cannot be inferred that low-level cultural capital is a cause of poor health. Instead, the causes for these two health outcomes need to be examined separately. Asymmetry in conditional effects indicates that a condition that functions in one configuration may not work or may exert the opposite effect in other configurations. This type of asymmetry relaxes the assumption of uniform causal effect in linear regression and enables a better account of the differences between cases and the configurational effects arising from the interdependencies among conditions. In addition, QCA applicability spans large, medium, and small sample studies, particularly demonstrating unique explanatory power with medium-sized samples that avoids both the case homogeneity pitfall of large-sample statistics and the generalizability limitations of small-sample case studies. QCA has gained extensive application across disciplines.

In public health intervention research, scholars have successfully applied QCA to analyze intervention impacts and health determinants (34). Given cultural capital’s conceptual complexity and diverse pathways through which it influences health, this study adopts fsQCA to explore configurational relationships between cultural capital and health. The fsQCA analyses were implemented using R software version 4.4.2.

### Variable calibration

The fsQCA methodology comprises three sequential steps: calibration, necessity analysis, and sufficiency analysis. While the mathematical computations in these procedures can be executed through R software, manual determination of appropriate thresholds remains essential. Scholar Pappas summarized the application of fuzzy-set fsQCA and provided a detailed step-by-step guide. This guide offers methodological support for subsequent studies that adopt the fsQCA method (35). Within this guide, a comprehensive summary of the thresholds involved in the three steps of fsQCA is presented. Therefore, we have chosen the corresponding thresholds based on this summary.

The initial calibration process transforms raw data into fuzzy-set membership scores (ranging from 0 = full non-membership to 1 = full membership, with 0.5 representing the crossover point) to quantify case affiliation levels in specific conditions or outcomes. This study classified variables into three distinct types: continuous (or quasi-continuous), multi-categorical, and binary. For continuous or quasi-continuous variables (age, income, embodied cultural capital), calibration thresholds were established at 0.9, 0.5, and 0.1 quantiles. Multi-categorical variables required differentiated thresholding: 5, 3, 1 for 5-point Likert scales and 6, 4, 2 for 7-point Likert scales, applied to self-rated health, exercise, and institutionalized cultural capital. Binary variables required no calibration. Comprehensive calibration outcomes are systematically presented in Table 1.

## Result

### Necessity analysis

The second step in fsQCA involves necessity analysis, which aims to examine whether individual conditions constitute necessary requirements for outcome occurrence (i.e., “the condition must be present when the outcome emerges”). The presence of a necessary condition implies that the outcome cannot be achieved without it, though its sole presence does not guarantee the outcome. This analysis yields two critical metrics: Consistency and Coverage. Following established research criteria, a condition is typically deemed necessary if it meets thresholds of Consistency ≥ 0.9 and Coverage ≥ 0.6.

Table 2 presents the necessity analysis results, with condition variables categorized into presence/absence states and healthy outcome as the target. The findings demonstrate that all seven condition variables exhibit consistency values below 0.9, indicating that neither their presence nor absence constitutes a necessary condition for achieving healthy outcomes.

**Table 2:** Analysis of necessary conditions for health.

Condition	Consistency	Coverage
Age	0.546	0.688
~Age	0.662	0.840
Gender	0.515	0.644
~Gender	0.485	0.621
Marriage	0.753	0.631
~Marriage	0.247	0.637
Income	0.692	0.823
~Income	0.530	0.716
Exercise	0.589	0.777
~Exercise	0.580	0.705
Institutionalized cultural capital	0.356	0.849
~Institutionalized cultural capital	0.758	0.653
Embodied cultural capital	0.652	0.822
~Embodied cultural capital	0.554	0.704

The tilde “~” represents negation.

### Sufficiency analysis

The third step of fsQCA involves sufficiency analysis, which constitutes the core component. This process identifies sufficient conditional combinations. The initial phase requires constructing a truth table (a statistical representation of all possible combinations between conditional variables and the outcome variable) while establishing three evaluation criteria: consistency threshold, frequency threshold, and PRI threshold. Based on comprehensive reviews of previous scholarly work, the following parameters were determined: consistency threshold ≥0.8; frequency threshold adjustable according to sample size while maintaining residual case proportion ≥75%; PRI threshold ≥0.7. This study adopted thresholds of 0.8, 30, and 0.7 respectively, with remaining cases totaling 5,032 (87 > 75%). Following truth table construction, logical remainders were processed to derive three solutions. The complex solution excludes all logical remainders, the parsimonious solution incorporates all remainders, while the intermediate solution requires researcher-defined directional constraints for selected remainders. Guided by prior summary on social capital-health relationships, parameter settings for the intermediate solution designated age as negative (0), income and exercise as positive (1), with other variables remaining bidirectional (−). Final interpretation prioritized the intermediate solution with parsimonious solution supplementation to distinguish core from peripheral conditions.

Table 3 presents sufficiency analysis results for seven conditional variables predicting health status. Nine configurations emerged as sufficient conditions (solution consistency = 0.882 > 0.8), revealing complex cultural capital-health relationships through multiple pathways. Six configurations (3,4,5,6,8,9) demonstrated raw coverage >20%, indicating stronger explanatory power and broader case coverage. Notably, neither cultural capital variable independently or jointly formed stand-alone configurations, requiring synergistic interactions with other variables. Based on cultural capital distribution patterns and status, configurations were categorized into three types: Coexistence (1), Single existence (2–7), and Non-existence (8,9).

**Table 3:** Configuration analysis for achieving health.

Conditional variables	Health								
	1	2	3	4	5	6	7	8	9
Age	**⊗**		**△**		**△**		**△**	**△**	**△**
Gender		●					●	●	
Marriage	**⊗**	●	▲	▲	●	▲		●	●
Income		●	▲	▲		▲	▲	▲	▲
Exercise	●	●		●					
Institutionalized cultural capital	▲	▲			**⊗**	**⊗**	**⊗**		**⊗**
Embodied cultural capital	▲		▲	▲	▲	▲	▲		
Consistency	0.949	0.915	0.930	0.915	0.923	0.917	0.945	0.893	0.929
Raw coverage	0.080	0.095	0.320	0.296	0.272	0.290	0.154	0.209	0.295
Unique coverage	0.066	0.003	0.011	0.007	0.040	0.016	0.020	0.006	0.028
Solution consistency	0.882								
Solution coverage	0.610								

▲ indicate high-level core conditions, △ indicate low-level core conditions; ● indicate high-level auxiliary conditions, **⊗** indicate low-level auxiliary conditions.

Table 4 presents the summary of health pathway types. In the coexistence type, high levels of institutionalized cultural capital and embodied cultural capital need to combine with low levels of age, low levels of marriage, and high levels of exercise to generate healthy outcome. The population characteristics corresponding to this pathway are unmarried young adults with high educational attainment, high cultural literacy, and consistent exercise routines. The Raw coverage of this configuration is only 0.08, indicating a limited number of cases meeting these criteria, indirectly reflecting the scarcity of individuals simultaneously achieving high educational attainment and cultural literacy. The single existence type exhibits the highest number of configurations, demonstrating multiple pathways for healthy outcome. Among these six configurations, embodied cultural capital assumes a critical role (appearing at high levels five times as core conditions), whereas institutionalized cultural capital plays a minor role (high levels only once, with low levels in the remaining three instances). This indicates that within single-dimensional cultural capital pathways for healthy outcome, the accumulation of cultural practices is more significant than educational credentials. Among other conditional variables, high levels of marriage and income appear most frequently, suggesting their pivotal roles in this type of pathways. The most representative example is configuration 3 (raw coverage = 0.320), where high embodied cultural capital combines with high marriage, high income, and low age to produce healthy outcome, corresponding to high-income married young adults engaged in regular cultural activities. In the non-existence type, both cultural capital variables become irrelevant, and even in configuration 9, the institutionalized cultural capital appears at low level. These two configurations reflect scenarios where cultural capital has no significant or negative impact on health. Health promotion pathways under these configurations require three concurrent conditions: high Marriage, high Income, and low Age, corresponding to high-income married young adults.

**Table 4:** Summary of health pathway types.

Type	Number	Key condition combinations	Representative group
Coexistence	1	high level institutionalized cultural capital (core) +high level embodied cultural capital (core) +high exercise +low level age +low level marriage	Unmarried young adults, high education and culture, active
Single existence	2–7	high level embodied capital (core) +high level income +high level marriage +low level age	Married young adults, high income, cultural participation
Non-existence	8–9	high level income (core) +low level age (core) +high level marriage +no or low level institutionalized cultural capital	Married young adults, high income, limited culture

The column of key condition combinations is used to present and explain representative configurations.

## Discussion

The study employed a configurational approach through fsQCA to unravel the complex pathways through which individual cultural capital influences health outcomes. Our findings reveal that cultural capital operates synergistically with socioeconomic and demographic factors, forming multiple causal configurations that collectively shape health status. This aligns with Bourdieu’s theoretical framework, which posits that cultural capital functions within a broader system of social stratification, interacting dynamically with other forms of capital to reproduce health inequalities. Notably, the absence of necessary conditions in the analysis underscores the complexity of health determinants, where no single factor is indispensable, but combinations of conditions generate outcomes through conjunctural causation.

The sufficiency analysis identified nine distinct pathways, categorized into three types: coexistence, single existence, and non-existence of cultural capital. The coexistence pathway (Configuration 1), characterized by high institutionalized and embodied cultural capital, low age, and frequent exercise, highlights the synergistic role of education and cultural engagement in health promotion. However, its low raw coverage suggests that such combination accounts for relatively little in Chinese population, reflecting structural barriers to simultaneous accumulation of educational credentials and cultural practices (36). This resonates with studies emphasizing the unequal distribution of cultural capital in transitional societies, where educational expansion coexists with persistent disparities in cultural participation (37). This challenge in operationalising and measuring the simultaneous possession of different capital forms is not unique to China, as noted in educational research across various international contexts (38).

In single-existence pathways, embodied cultural capital emerged as a central driver of health outcomes, appearing as a core condition in five configurations. This finding supports the argument that active cultural engagement—such as reading, attending events, or internet use—enhances health literacy, social connectivity, and psychological well-being, independent of formal education (39). This underscores the notion that the value of capital is not absolute but is realized through its translation and application within specific fields and social economies (40). The prominence of embodied capital in our health-related findings parallels distinctions made in moral philosophy, where active, context-specific engagement (analogous to moral action) can be a more direct driver of outcomes than decontextualized knowledge or credentials (analogous to moral thought) (41). For instance, Configuration 3 illustrates that high embodied cultural capital, combined with marriage, income, and youth, forms a robust pathway to health. This aligns with research linking cultural participation to stress reduction and improved social support (16). Conversely, institutionalized cultural capital (e.g., educational attainment) played a peripheral role, suggesting that in China’s rapidly evolving social landscape, informal cultural practices may outweigh formal credentials in mediating health behaviors (42, 43).

The non-existence pathways (Configurations 8 and 9) reveal scenarios where cultural capital is irrelevant or even inversely associated with health. Here, high income, marital stability, and youthfulness compensate for low cultural capital, underscoring the compensatory role of economic and social resources. This echoes studies in low- and middle-income countries, where economic capital often supersedes cultural capital in health determination (44). Furthermore, this compensatory role of non-cultural resources finds parallels in international literature. For instance, robust social capital has been shown to sustain life satisfaction and well-being among coaches even during the challenges of the COVID-19 pandemic (45), illustrating a similar mechanism where one form of capital can effectively offset the lack of others in different national and professional settings. Notably, Configuration 9 demonstrates that low institutionalized cultural capital coexists with favorable health outcomes when supported by financial stability and marital cohesion.

Our results also highlight the critical role of demographic factors. Age consistently appeared as a low-level condition across multiple configurations, indicating that youthfulness amplifies the health benefits of cultural and socioeconomic resources. This aligns with life-course perspectives, wherein younger individuals possess greater adaptability to leverage cultural capital for health optimization (46). Marriage and income further emerged as pivotal moderators, corroborating evidence that economic security and social support mediate the translation of cultural resources into health advantages (47).

Methodologically, this study demonstrates the utility of QCA in disentangling complex social determinants of health. Traditional regression models, which assume linearity and variable independence, fail to capture the synergistic and asymmetric relationships revealed here. For instance, while regression might identify cultural capital as a “net positive” predictor, QCA unveils its conditional effectiveness—dependent on specific combinations with age, income, or marital status. This advances theoretical frameworks by emphasizing equifinality: diverse pathways can yield similar health outcomes, necessitating tailored policy interventions.

## Limitations and future directions

First, it is important to note that the causal inferences drawn from this study are constrained by its cross-sectional design. The configurations identified represent associations between cultural capital, socio-demographic factors, and health outcomes at a single point in time. While fsQCA reveals complex pathways and necessary/sufficient conditions, longitudinal data are required to establish temporal precedence and strengthen claims of causality. Second, the operationalization of cultural capital via CGSS proxies may overlook nuanced dimensions, such as digital literacy or intergenerational transmission. Future research could incorporate mixed methods to enrich measurement validity.

## Conclusion

This study employed the method of QCA to explore how cultural capital configurations influence health in a Chinese population. Key findings underscore that:

1) Cultural capital influences health through multiple, equifinal pathways rather than in isolation.
2) Embodied cultural capital (cultural participation) is a more potent and consistent driver of good health than institutionalized cultural capital (education) in many pathways.
3) Socioeconomic (income) and demographic (age, marriage) factors interact with cultural capital, and can sometimes compensate for its lack.

Public health strategies should prioritize fostering inclusive cultural participation—e.g., through subsidized community arts programs, public library and museum access initiatives, and digital literacy training—to enhance embodied cultural capital among disadvantaged groups.

## Data Availability

The datasets presented in this study can be found in online repositories. The names of the repository/repositories and accession number(s) can be found below: The datasets analysed during the current study are available in the CGSS repository, http://www.cnsda.org/index.php?r=projects/view&id=65635422.
